# 724 Role of synthetic dermal matrix for reconstruction of complex non-graftable wound defects

**DOI:** 10.1093/jbcr/irac012.278

**Published:** 2022-03-23

**Authors:** Elizabeth Concannon, Marcus Wagstaff

**Affiliations:** Royal Adelaide Hospital, Princes Hill, Victoria; Royal Adelaide Hospital, Adelaide, South Australia

## Abstract

**Introduction:**

The aim of this study was to investigate the role of a synthetic dermal matrix, Biodegradable Temporising Matrix (BTM), for coverage of complex wounds. The authors defined complex wounds as wounds not amenable to reconstruction with skin grafting alone due to an inherent avascularity of the wound bed, such as the presence of exposed bone, tendinous or neural structures.

**Methods:**

A retrospective review of a prospectively maintained database of complex wounds as defined above was carried out. Clinical and operative notes were reviewed along with review of an extensive photographic database demonstrating wound healing progress using staged dermal matrix (BTM) and autologous skin graft reconstruction.

**Results:**

55 patients were identified who underwent staged dermal matrix and autologous skin graft reconstruction for complex wounds affecting a wide variety of patient demographics, treatment indications and body sites. Wound aetiology varied between burn injury, non-burn related trauma including degloving injury and infective complications. We discuss caveats relating to successful application of a dermal matrix, technique tips, prevention and management of complications.

Dermal substitutes play an integral role in providing biological wound cover for avascular wound beds which may otherwise require complex distant flap or microsurgical free flap reconstruction. BTM is a completely synthetic dermal matrix comprised of a 2-mm-thick sheet of biodegradable polyurethane foam bonded to a non-biodegradable polyurethane sealing membrane. Our department has developed significant expertise in the use of BTM throughout its development from initial animal studies through to recent human clinical trials. The synthetic composition of BTM does not require rapid neo-vascularisation for its integrity or survival. As such, two-stage BTM reconstruction has proven robustness in the face of unfavourable wounds compared with other popular dermal matrices, physiologically covering avascular structures, allowing for early graft take, expediting rehabilitation and mobilisation with excellent scar cosmesis and limited contracture formation.

**Conclusions:**

Dermal matrices such as BTM play an important role in complex wound healing, frequently achieving excellent results with a low complication profile. BTM has been used successfully in cases where biological matrices would not routinely be considered as demonstrated by this clinical series. It has provided a valuable alternative to free-tissue transfer in patients with significant co-morbidities, vascular insufficiency and/or those for whom long operations are undesirable.

